# Supplementary material for: 2D layered transport properties from topological insulator Bi2Se3 single crystals and micro flakes
Source: Sci Rep. 2016 Jun 7;6:27483. doi: 10.1038/srep27483 (PMC4895388; doi:10.1038/srep27483)
Supplement: Supplementary Information [file srep27483-s1.pdf]

2D layered transport properties from topological insulator Bi_2Se_3 single crystal and micro flakes

Olivio Chiatti[1], Christian Riha[1], Dominic Lawrenz[1], Marco Busch[1], Srujana Dusari[1], Jaime Sánchez-Barriga[2], Anna Mogilatenko[3], Lada V. Yashina[4], Sergio Valencia[2], Akin A. Ünal[2], Oliver Rader[2], and Saskia F. Fischer[1,*]

[1] Novel Materials Group, Humboldt-Universität zu Berlin, Newtonstraße 15, 12489 Berlin, Germany
[2] Helmholtz-Zentrum-Berlin für Materialien und Energie, Albert-Einstein-Straße 15, 12489 Berlin, Germany
[3] Ferdinand-Braun-Institut, Leibniz-Institut für Höchstfrequenztechnik, Gustav-Kirchhoff-Straße 4, 12489 Berlin, Germany
[4] Department of Chemistry, Moscow State University, Leninskie Gory 1/3, 119991 Moscow, Russia
[*] Correspondence and requests should be addressed to S.F.F. (email: sfischer@physik.hu-berlin.de)

Supplemental information

Fig. 1(a) shows a typical low-magnification transmission electron microscopy (TEM) image of a Bi_2Se_3 flake with an inhomogeneous thickness. As shown in the small inset of Fig. 1(a), the edges of the exfoliated flake run along two sets of prismatic planes: $\{11\bar{2}0\}$ (gray) and $\{1\bar{1}00\}$ (black). Fig. 1(b) presents a high angle annular dark-field scanning transmission electron microscopy (HAADF-STEM) image of the Bi_2Se_3 flake. An abrupt intensity change corresponds to a step-like increase of the flake thickness.

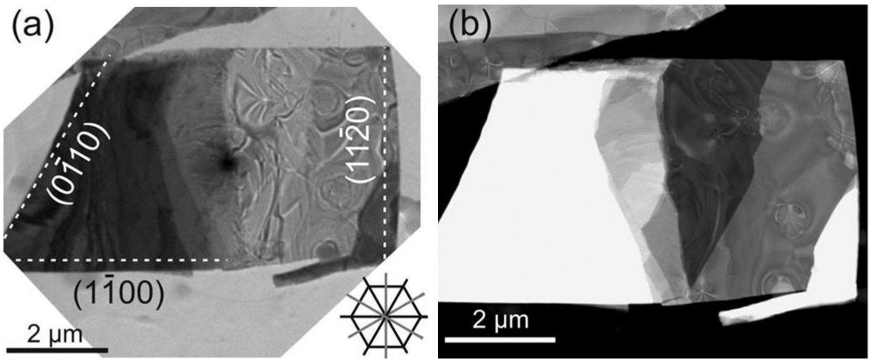

Figure 1. (a) Low-magnification TEM image of a Bi_2Se_3 flake with an inhomogeneous thickness. (b) HAADF-STEM image revealing the presence of steps at the flake surface.

In order to search for adhered Bi_2Se_3 micro flakes, the samples were scanned using optical microscopy. The flakes were examined using a combination of confocal microscopy, AFM, SEM, HRTEM and EDX. Firstly, we used confocal microscopy to characterize the thickness homogeneity of the flakes. This type of measurements are important because homogeneous

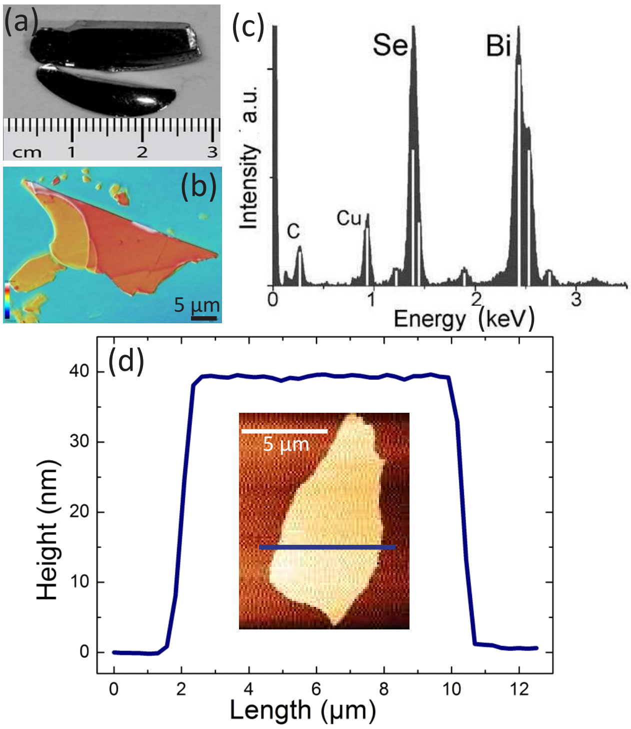

Figure 2. (a) Image of bulk Bi_2Se_3 crystal. (b) Confocal microscope image of an inhomogeneously thick Bi_2Se_3 micro flake. (c) EDX spectrum used for the compositional analysis. C and Cu peaks stem from the carbon-coated copper-grid. (d) AFM image of a 39 nm thick Bi_2Se_3 micro flake.

thickness is crucial to obtain consistent information from transport measurements. Fig. 2(b) shows an example of a confocal microscopy image of an inhomogeneous flake. Different thicknesses, probably appearing during the cleavage procedure, are clearly seen as slightly different contrast. Once homogeneous flakes were selected, their thickness was measured with AFM (TopoMetrix TMX 2000 Explorer SPM) in non-contact mode under ambient conditions.

It is known that the elemental composition plays an important role in the transport mechanism of TIs. The chemical composition of the flakes was studied by EDX spectroscopy (see Fig. 2(c)). The Bi/Se ratio was calculated using theoretical Cliff-Lorimer coefficients. This analysis confirms that the stoichiometry of the flakes is preserved after mechanical exfoliation within 1−2% accuracy. However, we emphasize that the defect chemistry in Bi_2Se_3 is dominated by charged Se vacancies which act as electron donors leading to *n*-type behavior. This will result in a modification of the transport properties depending on the amount of Se vacancies. Using EDX it is not possible to obtain accurate quantitative information on the small level of Se vacancies contributing to conducting carriers in Bi_2Se_3 which is significantly smaller than 1% level.

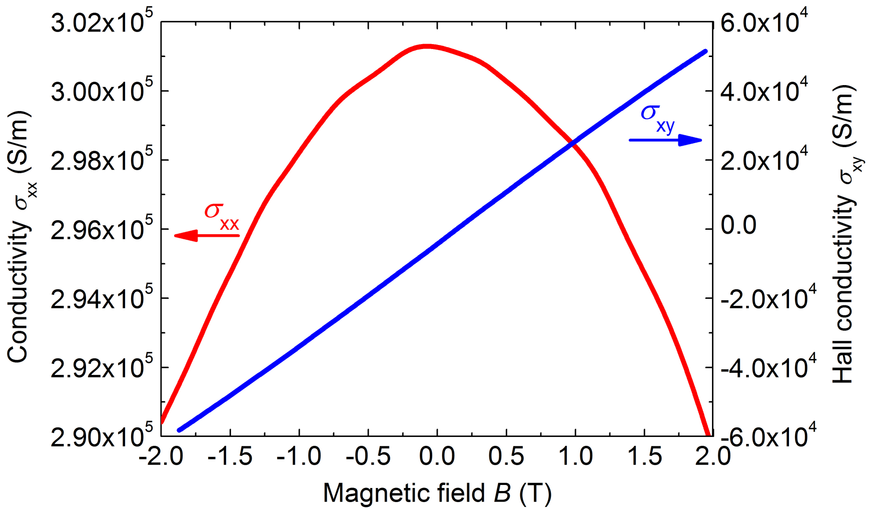

Figure 3. Conductivity σ_{xx} (red curve, left axis) and Hall conductivity σ_{xy} (blue line, right axis) vs magnetic field B of the Bi_2Se_3 bulk crystal with a thickness of $t = 110$ μm at $T = 4.1$ K.

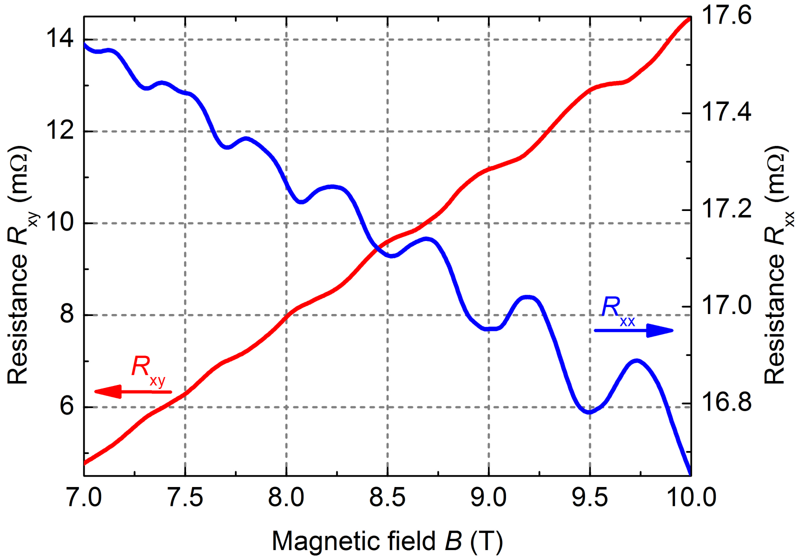

Figure 4. Resistances R_{xy} (red curve, left axis) and R_{xx} (blue line, right axis) vs magnetic field B of a further Bi_2Se_3 macro flake. SdH oscillations in R_{xx} indicate the occurence of Landau levels. The quantization in R_{xy} is clearly visible at magnetic field B of around 8.5, 9.0 and 9.5 T.
